# Comparative Evaluation of Retentive Properties of Two Compatible Ball Attachments in Mandibular Implant-Retained Overdentures: An In Vitro Study

**Published:** 2018-03

**Authors:** Maryam Memarian, Simindokht Zarrati, Sedigheh Karimi, Mehran Bahrami

**Affiliations:** 1 Associate Professor, Dental Research Center, Dentistry Research Institute, Tehran University of Medical Sciences, Tehran, Iran; Department of Prosthodontics, School of Dentistry, Tehran University of Medical Sciences, Tehran, Iran; 2 Assistant Professor, Dental Research Center, Dentistry Research Institute, Tehran University of Medical Sciences, Tehran, Iran; Department of Prosthodontics, School of Dentistry, Tehran University of Medical Sciences, Tehran, Iran; 3 Prosthodontist, Private Practice, Tehran, Iran; 4 Assistant Professor, Department of Prosthodontics, School of Dentistry, Tehran University of Medical Sciences, Tehran, Iran

**Keywords:** Dental Prosthesis, Implant-Supported, Overdenture, Dental Prosthesis Retention

## Abstract

**Objectives:**

The retentive properties of implant-retained overdentures (IRO) may be influenced by the type of attachments. The aim of this research was to compare the retention of two dental implant systems with compatible ball attachments, namely Straumann® system (SS) and Rhein83 SRL system (RS) after fatigue testing.

**Materials and Methods:**

Two laboratory models consisting of two parallel Straumann® fixtures at a distance of 22 mm were prepared. Five pairs of each systems’ ball attachments were examined (n=5). The samples were soaked in artificial saliva. The retention strength values (RSV) were recorded before the fatigue test and after 1100, 2200, 3300, 4400, and 5500 insertion and removal cycles at a speed of 51 mm/minute with a 50-N load cell in a universal testing machine. The data were analyzed by repeated measures analysis of variance (ANOVA) followed by independent sample t-test with Bonferroni corrections.

**Results:**

There was a decrease in the RSV in both systems after 5500 cycles of insertion and removal. There was a significant statistical difference between the RSV of the normal Sphero Block of the RS (17.52±0.68 N) and that of the Spare Lamella retention inserts of the SS (19.72±0.74 N, P=0.001).

**Conclusions:**

Although the RSVs of the RS and SS were almost similar before the fatigue test, as the number of insertion and removal cycles increased, the RSV decreased more significantly in the RS compared to the SS.

## INTRODUCTION

Nowadays, many different yet compatible implant systems are available as some manufacturers try to copy the pioneer and costly implant systems. Mandibular implant-retained overdentures (IRO) supported by two implants are often considered as the standard treatment for edentulous patients [1,2]. Various unsplinted attachments are available for IROs.

The key component of such attachment systems is the matrix which has been reported to be particularly weak in ball attachments and is in need of constant repair and adjustment [3,4]. Frequent repairs and maintenance of the matrix are challenging in the clinical setting [5–7]. The type of the attachment system plays a key role in the retention strength [8]. Compatible abutments of different implant systems may be used interchangeably to decrease the expenses. It is necessary to evaluate the retention strength values (RSV) of these abutments since a proper retention of attachments improves the patient satisfaction. This study was based on the controversial question that whether such compatible abutments cause long-term adverse effects due to the differences in their surface designs, dimensions, shapes, and materials compared to those of the original systems.

The differences could be associated with patent issues and manufacturing processes that may hinder the precise replication of the components. Some studies have suggested that it is better to avoid substituting the original components with the compatible ones since they may cause significant micromovements and leakage, and the components may not completely match [9–13]. However, if the designs of compatible abutment joints are carefully matched with the original implant connections, these problems may be negligible. Some other studies have explained that there were no significant differences between some of the compatible abutments and their original counterparts [14–17].

The ball attachments of the Rhein83 SRL system (RS) are compatible with the Straumann® dental implant system (SS). As the RS is less expensive than the SS, many clinicians and technicians prefer to use the RS. The aim of this study was to compare the RSVs in the RS and SS using compatible abutments. The null hypothesis was that there would be no significant differences between the RS and SS with regard to the RSVs.

## MATERIALS AND METHODS

**Fig. 1:**
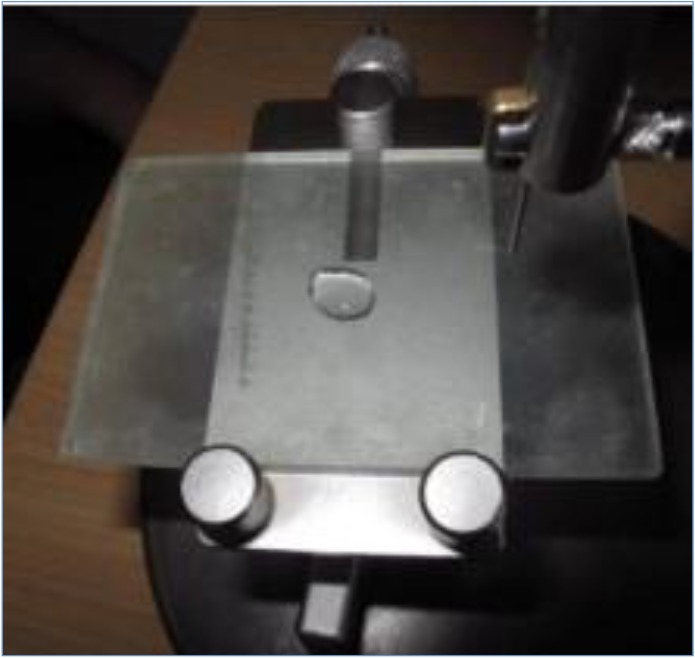
The water-drop test

**Fig. 2:**
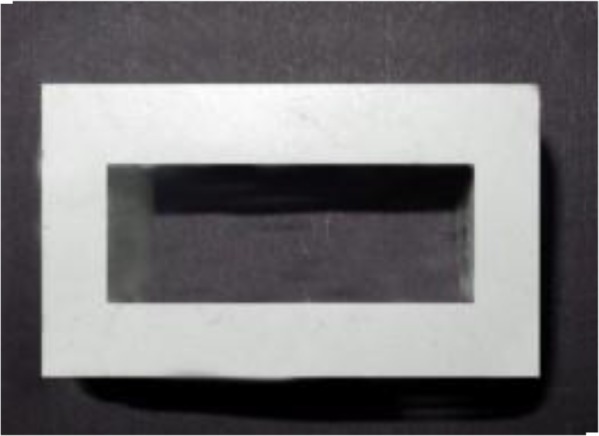
The prepared cube

**Fig. 3:**
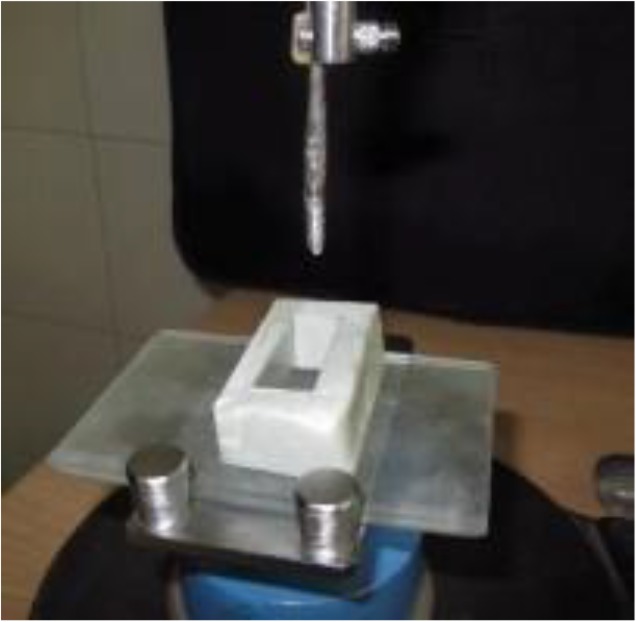
Insertion of implants by using the surveyor

A slab was placed on the table of a surveyor (BEGO, Paraflex precision surveyor, Kodent Dental Supply, Renton, Washington, USA), parallel to the horizon by using the water-drop test (Fig. 1). A cube of 20 mm height, 31 mm length and 13 mm width was made on the slab by using a heavy body silicone impression material (Speedex putty, Coltene/Whaledent Inc., Altstätten, Switzerland) (Fig. 2). Two fixtures with the diameter of 4.1 mm and length of 10 mm (ITI, Straumann® Holding AG Co., Basel, Switzerland) were cemented on the slab parallel to each other by using a cyanoacrylate adhesive (Benson polymers Ltd., New Delhi, Delhi, India) (Fig. 3). They were adjusted by using the vertical arm of the surveyor. The distance between these fixtures was 22 mm.

**Fig. 4:**
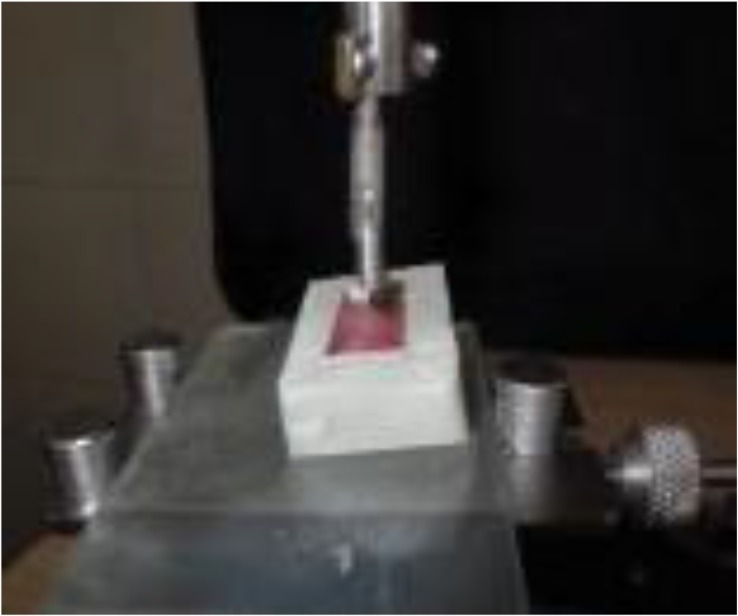
The cubes were filled with acrylic resin

The cube was filled with a self-curing clear acrylic resin (Meliodent, Heraeus Kulzer GmbH, Germany) (Fig. 4). The procedures were repeated to fabricate two identical laboratory IRO models.

In order to evaluate the RSVs in the IRO models, two ball and socket attachment systems were used; one from the SS (Retentive Anchor; Elite/Titanium male: Height=3.2mm, Diameter=3.6mm, and Titanium female: Height=3.4mm, ITI, Straumann® Holding AG Co., Basel, Switzerland), and the other one from the RS (normal Sphero Block abutments; Stainless steel male: Height=4.6mm, Diameter=3mm, and Titanium-nitrate-coated female: Diameter=2.5mm, Rhein83 SRL, Bologna, Italy). Five pairs of each system’s ball attachments were examined (n=5).

**Fig. 5:**
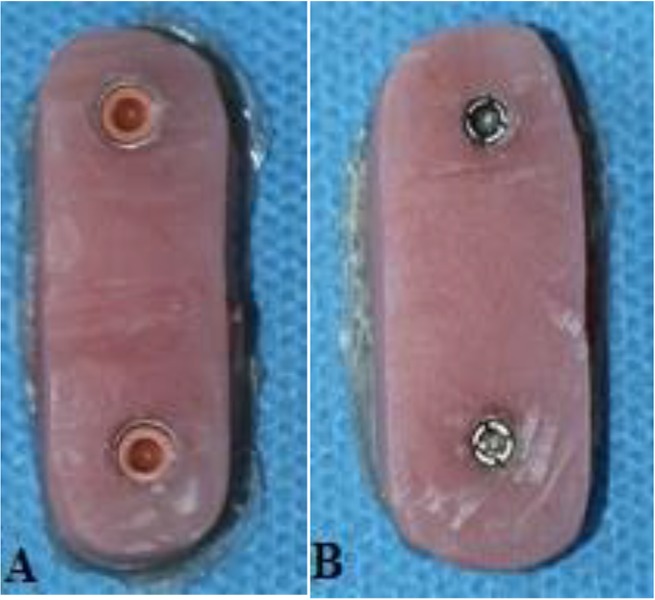
Acrylic molds containing the implant-retained overdenture (IRO) housings of (A) the Rhein83 SRL system (RS) and (B) the Straumann® system (SS)

**Fig. 6:**
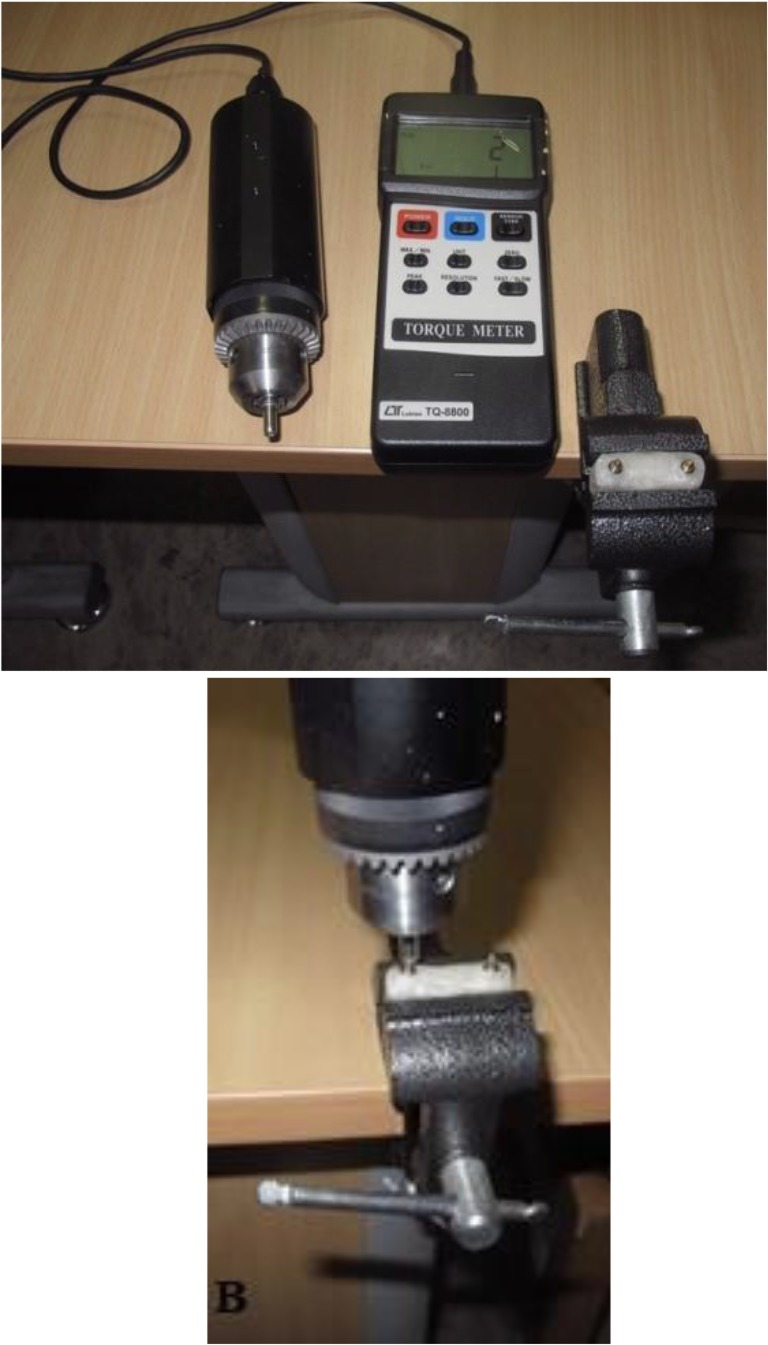
The electronic-torque-meter (A) is tightening the abutments (B)

**Fig. 7:**
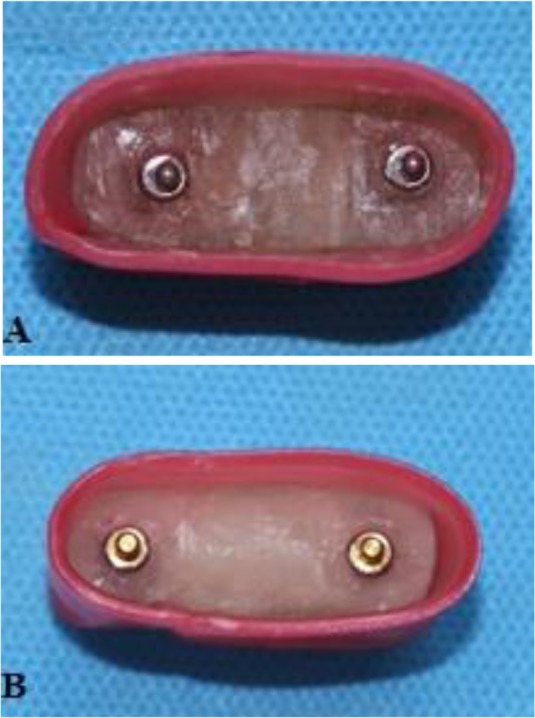
(A) Boxing of the acrylic mold with two Straumann® fixtures. (B) Elliptical matrices are placed on the retentive anchors

**Fig. 8:**
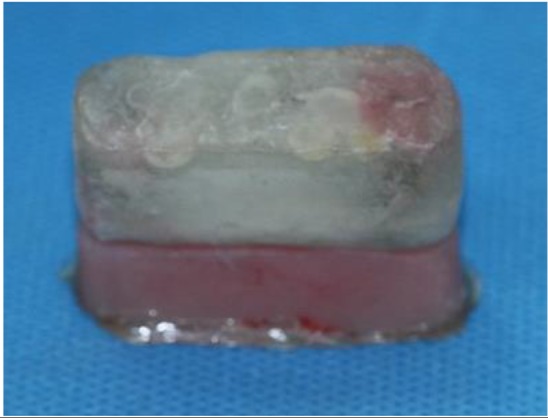
The prepared two-part model

Afterwards, two ball abutments of the SS (Retentive Anchors, ITI, Straumann® Holding AG Co., Basel, Switzerland) and two normal Sphero Block abutments of the RS (Rhein83 SRL, Bologna, Italy) were placed on the fixtures in the acrylic molds by using special wrenches. Two Straumann® elliptical matrices, containing Spare Lamella retention inserts, were placed on the retentive anchor abutments. In the second cylinder, containing the Rhein83 SRL ball attachments, two normal stainless steel housings with two normal retentive pink caps were placed on the normal Sphero Blocks (Fig. 5). According to the manufacturer, a torque meter (LT, Lutron TQ-8800, Taipei, Taiwan) was used to reach a 35-newton-centimeter (Ncm) torque (Fig. 6). The abutments were re-tightened to the same torque after 10 minutes to achieve the desirable preload according to the protocol applied by Dixon et al [10] and Breeding et al [11]. The abutments were tightened again to the prescribed torque as dictated by the standards of Straumann® ball attachments. After inserting the Spare Lamella retention, the wrench was again used to rotate the lamella for 360 degrees in a clockwise manner to achieve the level of retention strength approximately equal to that of the pink caps of the ball attachments of the RS. The abutments’ necks were blocked-out with a thin layer of a modeling wax (Cavex Holland, BV, Haarlem, Netherlands). Afterwards, the acrylic models were boxed and filled with the self-curing acrylic resin to form the upper portion of the model (Fig. 7). The excess acrylic resin was removed from the areas surrounding the attachments. Next, the two parts of the blocks were assembled for fatigue testing (Fig. 8).

**Fig. 9:**
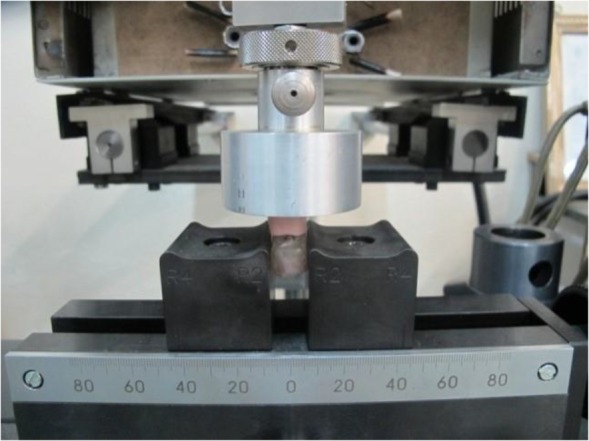
Model in the universal testing machine

The samples were floated in one liter of artificial saliva (Sodium bicarbonate (NAHCO_3_), 200 mmol/l; Phosphorus (P), 30 mmol/l; Calcium (Ca), 1.5 mmol/l; pH=7), which was prepared by the assistance of the Dental Biomaterial Synthetic Laboratory (School of Dentistry, Tehran University of Medical Sciences, Tehran, Iran) at room temperature. Afterwards, the samples were subjected to fatigue testing in a universal testing machine (Zwick/Roell, Zwick GmbH&Co. KG, Ulm, Germany) (Fig. 9) using 1100, 2200, 3300, 4400, and 5500 insertion and removal cycles [12,13, 18]. The RSVs were measured three times to calculate the mean value which was considered as the primary or baseline retention strength. The mean value was used as a reference number for comparisons to the RSVs at the end of each cycle [2,19]. The machine was set at the pace of 51 mm/minute. A 50-N load cell with the mobility of 2.5 mm was attached to the upper part of the model which contained the housing [20]. The numbers corresponded to the pace of placement and removal of an IRO on/from the attachments during a 5-year period [12,13, 18]. The RSVs were recorded after each cycle of the fatigue test [19] and were considered as the maximum forces applied before the total separation of the attachments. Therefore, the RSV is the maximum force needed to dislodge the overdenture (the upper portion of the sample) from the attachments (the lower portion of the sample).

Each cycle of the fatigue test took 5 seconds, and each sample required 8 hours to be fully examined. All the mentioned steps were repeated for the five pairs of RS attachments and the five pairs of SS attachments. There were no traces of loosening or mobility in the balls or the housings, neither during nor at the end of the process.

The continuous variable (RSV), as a dependent variable, was reported in mean and standard deviation (SD). In order to test the effects of independent variables (attachment systems and cyclic loading) on the mentioned dependent variable, two-way analysis of variance (ANOVA) was used. However, because of the significant interaction between the variables, the statistical analysis strategy was changed, and in each cyclic loading, the effect of the RS and SS on the dependent variable (RSV) was evaluated by independent student’s t-test. In order to compare the effect of cyclic loading on the RSV at different stages of the fatigue test (6 stages: 0, 1100, 2200, 3300, 4400, and 5500 cycles) repeated-measures ANOVA was used followed by Bonferroni corrections for pairwise comparisons. P-values lower than 0.05 were considered statistically significant.

## RESULTS

**Fig. 10:**
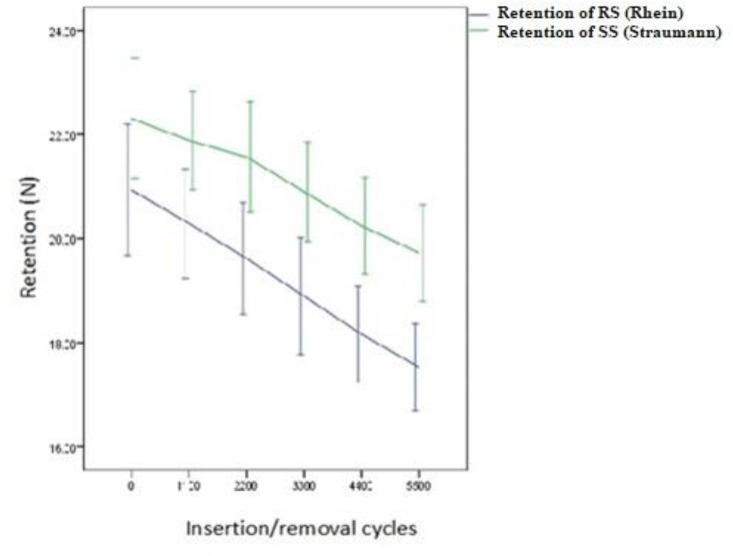
Fatigue test results of the studied groups during different insertion and removal cycles; the graph shows the mean retention forces (N) with 95% confidence intervals (CI) of the data

There were significant reductions in the RSVs of the RS after each cycle. On average, the primary (baseline) retention force of the RS was 20.94±1.01 N; however, it reduced to 20.29±0.86 N after 1100 cycles. The same significant descending trend was observed during 2200, 3300, 4400, and 5500 cycles (P<0.001). Likewise, the RSVs in the SS significantly declined. The baseline RSV was 22.31±0.92 N; however, it reduced to 21.87±0.76 N after 1100 cycles. The same significant reductions were seen during 2200, 3300, 4400, and 5500 cycles (P<0.001, Fig. 10). Table 1 shows the results of independent student’s t-test in comparing the RSVs of the two attachment systems categorized by the insertion and removal cycles.

**Table 1.** Retention strength values (RSVs) during different insertion and removal cycles (Independent student’s t-test)

**Number of cycles**	**Number of samples**	**Retention strength (N) of RS Mean±SD**	**Retention strength (N) of SS Mean±SD**	**P-value**
(0)	5	20.94±1.01	22.31±0.92	0.056
1100	5	20.29±0.86	21.87±0.76	0.014
2200	5	19.62±0.86	21.57±0.86	0.045
3300	5	18.98±0.90	20.90±0.77	0.005
4400	5	18.16±0.74	20.24±0.74	0.002
5500	5	17.52±0.68	19.72±0.74	0.001

RS=Rhein83 SRL system, SS=Straumann® system (SS), SD=Standard Deviation

## DISCUSSION

In the present study, the RSVs of both attachment systems were evaluated prior to the experiment, and no significant differences were detected. Therefore, the null hypothesis was true prior to the fatigue test. The initial RSV of the SS (22.31±0.92 N) was higher than that of the RS (20.94±1.01 N). The RSVs of both systems decreased after 1100 cycles. The RSV of the RS (20.29±0.86 N) declined more significantly than that of the SS (21.87±0.76 N). As the number of the cycles increased, the RSVs of both systems continued to decrease. The SS showed significantly higher RSVs than the RS during the insertion and removal cycles. Therefore, the null hypothesis was rejected after the fatigue test. After 5500 cycles, the RSVs were reported to be 19.72±.074 N in the SS and 17.52±0.68 N in the RS. The RS components may not perfectly fit into the original fixture’s interface causing microgaps and micromovements which can result in the reduction of RSVs during the fatigue test.

It has been suggested that only the plastic parts of the attachments are prone to fatigue and friction, while the metallic parts remain unchanged [20]. Therefore, a proper attachment system enhances the longevity and retention of the IRO and increases the patient satisfaction [1,8]. Some invitro studies have reported an increase in the RSV of IRO attachments after cyclic loading.

They have related this finding to the increased surface roughness and to the deformation of the components [19,21, 22]. In an in-vitro study, Zarati et al [23] evaluated vertical microgaps and torque loss in two ball attachment systems in IROs. They concluded that these problems were statistically more frequent in the RS than in the SS [23].

In order to investigate the necessary RSV for IROs, most of the researchers rely on the retention strength of 3.5 to 7 N according to Becker [22] and Korber [24], which is sufficient for the retention of a Kennedy class I removable partial denture [19]. However, this retention strength should not be considered as a desirable criterion to measure the retention of different implant attachment systems since the RSVs in IROs exceed 7 N [12,25]; similar results were found in the current study.

In a clinical retrospective survey, Fromentin et al [5] investigated the gold matrices of Straumann® ball attachments after being used for 8 years. They showed that one year of clinical use of the IRO had an adverse effect on the matrix retention. They also expressed that there was a considerable reduction in the matrix thickness, especially on the top of the holding lamella after 8 years of clinical use [5]. Similarly, in the present in-vitro study, reduced RSVs were observed at the end of the first year in both systems.

Gamborena et al [18] expressed a direct correlation between the number of cycles and the reduction in the RSV of the ERA® plastic attachments (APM-Strengold). In the present study, the RSV reduction was more significant in the RS with a plastic cap. This might be due to the different compositions of the plastic components, different sizes and forms of the caps, and the differences in the methods of the two studies.

In an in-vitro study, Walton and Ruse [26] compared the RSVs of three bar and clip attachments of IROs supported with two implant analogs before and after fatigue testing. According to their report, the RSV reduction was 12% after 5500 cycles, which corresponds to 3 years of clinical use. The findings of the present study were similar to those of Walton and Ruse [26]. However; according to some other studies, 5500 cycles correspond to 5 years of clinical use [12,13, 18].

Epstein et al [27] observed a reduction in the RSVs of plastic and metallic components of intraradicular attachment systems after 2000 cycles. The increased surface roughness of the components and the intraoral conditions could deform the plastic components, reduce the RSVs, and even trigger fractures [27].

Setz et al [28] observed a slight retention loss in most of the attachment systems during 15000 cycles. As the reduction was slight in most of the systems, the authors suggested that axial forces could not imitate the clinical conditions.

The deformation of the polymeric components and heat expansion in the absence of liquids or a salivary substitute could be among the reasons of the slight reduction of the RSVs in the mentioned research since a reduced RSV is a common clinical complication.

Pigozzo et al [12] compared the RSVs of four different bar and clip attachments of IRO systems after 5500 cycles in artificial saliva. They examined two polymer clips (Conexão and Sterngold Hader) and two metal clips (3i Gold Hader and S.I.N Clipo). They expressed that the metal components of the S.I.N implant system had a lower retention when compared to the RSVs of the other two plastic systems.

Nonetheless, the S.I.N system has the smallest components among the studied bar and clip attachments. The S.I.N system includes the thinnest component of the holding clip, the shortest clip, and the smallest bar diameter [12]. This was not in agreement with the findings of the present study which indicated that the metallic attachments in the SS had higher RSVs during fatigue testing.

In a retrospective case series, Hsu et al [14] concluded that compatible computer-aided design/ computer-aided manufacturing (CAD-CAM) titanium abutments can be used for posterior single-implant tooth replacement. However, they expressed that screw loosening and decementation during a 6-year follow-up may be related to the type of cement and abutments [14].

In an in-vitro study, Lee et al [15] evaluated the detorque values of four compatible abutment screws. They concluded that there were no significant statistical differences among the studied screws in terms of the torque loss values. In the current study, the RSVs were examined prior to and after the fatigue test. According to the results, there were significant statistical differences between the SS and RS after each cycle of the fatigue test.

Dellow et al [16] evaluated the implant-abutment-interface fit of the components of four compatible dental implant systems by using scanning electron microscopy (SEM). They concluded that some of the compatible abutments are interchangeable. Interestingly, their results showed that some of the studied abutments could fit as perfectly as the components of the original system. However; the results of the current study showed that the RSVs of the SS (as the original component) were higher than those of the RS (as the compatible component).

In an in-vitro study, Cashman et al [17] analyzed the post-fatigue-reverse-torque values (PFRTV) in Straumann® abutments and compared them to those of one of their compatible abutments. Their results showed that there was no statistically significant decrease in the PFRTV of the studied abutments. In our study, the RSVs were examined in the SS and its compatible counterpart, i.e. the RS. The results of our study showed that there were no significant statistical differences in the RSVs of the RS and SS prior to the fatigue test. However, after each cycle of the fatigue test, which is more similar to the clinical conditions, the RSVs of the RS were significantly decreased compared to those of the SS.

Berberi et al [29] showed that substitution of original abutment components with the compatible ones caused significant differences in the leakage at the implant-abutment connection; therefore, this substitution should be avoided. In another study, Berberi et al [30] indicated that as the compatible abutments would not perfectly fit into the internal connections of OsseoSpeed™ implants, the leakage level of the final assembly could increase. Mattheos et al [9] compared the morphological micro-features of two commercially available implant-abutment joints by finite element analysis (FEA). They investigated the correlations between the micromorphology and functional complications and proposed that compatible abutments might have significant morphological differences with the original ones. The differences found in the cross-sectional geometry could lead to more significant differences in the quality and extent of the overall contact areas. They implied that these differences may jeopardize the long-term stability of the prosthesis [9].

Alves da Cunha Tde et al [31] showed that there was a significant misfit between Procera zirconia abutments and different implant systems from other manufacturers. In another study, Berberi et al [32] expressed that the use of compatible abutments resulted in a significant amount of micromovement. According to these studies, the different RSVs of the SS and RS may be explainable. There might be some misfit and/or micromovement between the original fixtures and the RS components, which may cause a significant reduction in the RSVs of the RS compared to those of the SS after cyclic loading. One of the limitations of the current study is that the oral conditions including the presence of saliva, chemical effects of foods and drinks, and mouth temperature, which affect the long-term retention of the attachment systems, cannot be evaluated because of the in-vitro design of the study. In the present in-vitro study, the RSVs of the SS and RS were evaluated during the placement and removal of the prosthesis. Evaluating the other functional forces (rather than placement and removal) that are exerted on the attachments during normal functions are suggested in future studies.

Another limitation was that it was almost impossible to make the RSVs of both systems precisely equal prior to cyclic loading. The following method was the only way to approximately equalize the RSVs: the RSV of the pink caps of the RS is 800–950g, while the initial RSV of the Spare Lamella inserts of the SS is 200–1400g according to the manufacturers’ manual. The RSV can be adjusted in the range of 200 to 1400g by using a screwdriver (Art. No. 046.154, ITI). By a 90-degree clockwise rotation of the Spare Lamella, the retention was increased by up to 700g according to the manual.

Nonetheless, the RSVs of the two systems were not the same as each other before the fatigue test. According to the results of the present in-vitro study and by considering the RSVs, the use of the RS components instead of the original SS components is not recommended. Although there were no significant statistical differences before the fatigue test, the RSVs of the RS were significantly lower compared to those of the SS after the fatigue test.

## CONCLUSION

By considering the limitations of the present invitro study, the following conclusions were drawn:

1. The two studied attachment systems (SS and RS) did not have any significant differences in the RSVs prior to fatigue testing.
2. After 1100 insertion and removal cycles, the RSVs significantly reduced in both systems.
3. As the number of the cycles increased, the reduction in the RSVs continued in both systems; however, the RSVs reduced more significantly in the RS compared to the SS.
